# Effects of once-weekly semaglutide vs once-daily canagliflozin on body composition in type 2 diabetes: a substudy of the SUSTAIN 8 randomised controlled clinical trial

**DOI:** 10.1007/s00125-019-05065-8

**Published:** 2020-01-02

**Authors:** Rory J. McCrimmon, Andrei-Mircea Catarig, Juan P. Frias, Nanna L. Lausvig, Carel W. le Roux, Desirée Thielke, Ildiko Lingvay

**Affiliations:** 1 School of Medicine, University of Dundee, Ninewells Hospital and Medical School, Dundee, DD1 9SY UK; 2 Novo Nordisk A/S, Vandtårnsvej, Søborg, Denmark; 3 National Research Institute, Los Angeles, CA USA; 4 Diabetes Complications Research Centre, University College Dublin, Dublin, Ireland; 5 Department of Internal Medicine, UT Southwestern Medical Center, Dallas, TX USA

**Keywords:** Body composition, Canagliflozin, Fat mass, Glucagon-like peptide receptor agonists, Randomised controlled trial, Semaglutide, Type 2 diabetes, Weight

## Abstract

**Aims/hypothesis:**

Intra-abdominal or visceral obesity is associated with insulin resistance and an increased risk for cardiovascular disease. This study aimed to compare the effects of semaglutide 1.0 mg and canagliflozin 300 mg on body composition in a subset of participants from the SUSTAIN 8 Phase IIIB, randomised double-blind trial who underwent whole-body dual-energy x-ray absorptiometry (DXA) scanning.

**Methods:**

Adults (age ≥18 years) with type 2 diabetes, HbA_1c_ 53–91 mmol/mol (7.0–10.5%), on a stable daily dose of metformin (≥1500 mg or maximum tolerated dose) and with an eGFR ≥60 ml min^−1^ [1.73 m]^−2^ were randomised 1:1 to semaglutide 1.0 mg once weekly and canagliflozin placebo once daily, or canagliflozin 300 mg once daily and semaglutide placebo once weekly. Body composition was assessed using whole-body DXA scans. The study participants and investigator remained blinded throughout the trial, and quality of DXA scans was evaluated in a blinded manner. Change from baseline to week 52 in total fat mass (kg) was the confirmatory efficacy endpoint.

**Results:**

A subset of 178 participants (semaglutide, *n* = 88; canagliflozin, *n* = 90) underwent DXA scanning at screening and were randomised into the substudy. Of these, 114 (semaglutide, *n* = 53; canagliflozin, *n* = 61) participants had observed end-of-treatment data included in the confirmatory efficacy analysis. Of the 178 participants in the substudy, numerical improvements in body composition (including fat mass, lean mass and visceral fat mass) were observed after 52 weeks with both treatments. Total fat mass (baseline 33.2 kg) was reduced by 3.4 kg and 2.6 kg with semaglutide and canagliflozin, respectively (estimated treatment difference: –0.79 [95% CI −2.10, 0.51]). Although total lean mass (baseline 51.3 kg) was also reduced by 2.3 kg and 1.5 kg with semaglutide and canagliflozin, respectively (estimated treatment difference: −0.78 [−1.61, 0.04]), the proportion of lean mass (baseline 59.4%) increased by 1.2%- and 1.1%-point, respectively (estimated treatment difference 0.14 [−0.89, 1.17]). Changes in visceral fat mass and overall changes in body composition (assessed by the fat to lean mass ratio) were comparable between the two treatment groups.

**Conclusions/interpretation:**

In individuals with uncontrolled type 2 diabetes on stable-dose metformin therapy, the changes in body composition with semaglutide and canagliflozin were not significantly different. Although numerical improvements in body composition were observed following treatment in both treatment arms, the specific impact of both treatments on body composition in the absence of a placebo arm is speculative at this stage.

**Trial registration:**

ClinicalTrials.gov NCT03136484.

**Funding:**

This trial was supported by Novo Nordisk A/S, Denmark.

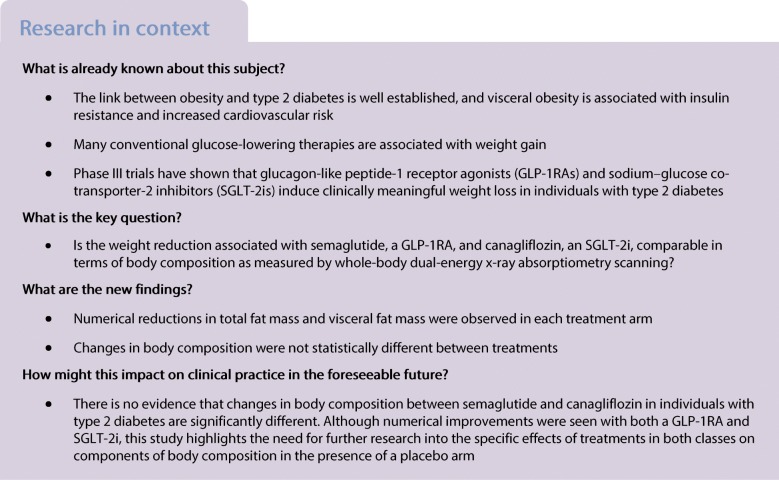



## Introduction

The association between obesity and type 2 diabetes is well established [1, 2]. Intra-abdominal or visceral obesity in particular is associated with insulin resistance [3, 4] and an increased risk for developing cardiovascular disease [4, 5]. In addition to providing recommendations for achieving glycaemic control, current type 2 diabetes guidelines emphasise the importance of weight loss through lifestyle changes, surgical interventions or medications [6, 7]. While some conventional glucose-lowering medications contribute to weight gain [2], newer agents – including those from the glucagon-like peptide-1 receptor agonist (GLP-1RA) and sodium–glucose co-transporter-2 inhibitor (SGLT-2i) classes – have favourable effects on body weight [2]. This is an important feature, because weight gain can lead to non-compliance with therapy [8].

Semaglutide is a GLP-1RA approved for the subcutaneous, once-weekly treatment of type 2 diabetes [9, 10]. The efficacy and safety of once-weekly semaglutide have been established in the SUSTAIN (Semaglutide Unabated Sustainability in Treatment of Type 2 Diabetes) clinical trial programme across the continuum of care in individuals with type 2 diabetes vs placebo and a range of comparators (SUSTAIN 1–5 and 7–10) [11–19] and in a cardiovascular outcomes trial (SUSTAIN 6) [20]. Canagliflozin is an SGLT-2i approved for the oral, once-daily treatment of type 2 diabetes [21]. The efficacy and safety of canagliflozin 100 mg and 300 mg have been demonstrated in an extensive clinical development programme that included cardiovascular and renal outcomes trials (CANVAS, CANVAS-R and CREDENCE) [22, 23]. Both semaglutide and canagliflozin are associated with substantial reductions in body weight. Across the SUSTAIN 1–10 trials, semaglutide has demonstrated significantly greater weight loss vs all comparators, with absolute change in body weight ranging from −3.8 to −6.5 kg with semaglutide 1.0 mg [11–20]. Canagliflozin has consistently demonstrated weight reductions of 2.5 to 4.7 kg in clinical trials [24].

Recently, a Phase IIIB randomised double-blind trial (SUSTAIN 8) compared the effects of once-weekly semaglutide 1.0 mg vs once-daily canagliflozin 300 mg on glycaemic control and weight management in participants with uncontrolled type 2 diabetes on a background of metformin therapy [17]. Treatment with semaglutide resulted in a mean change in HbA_1c_ from baseline to week 52 of −16.2 mmol/mol (−1.5%-point) compared with −10.4 mmol/mol (−1.0%-point) for canagliflozin (*p* < 0.0001), and a weight loss of −5.3 kg vs −4.2 kg, respectively (*p* = 0.003).

The magnitude of weight loss demonstrated in the SUSTAIN 8 trial would be expected to result in positive changes in body composition; however, as with any glucose-lowering agent, it is important to evaluate whether this weight loss has an adverse impact on the ratio of fat to lean mass.

To date, whether the effects on weight loss associated with the GLP-1RA semaglutide, and the SGLT-2i canagliflozin, are comparable in terms of total, lean and visceral fat have not yet been evaluated. This study compared body composition components in a subset of participants from SUSTAIN 8 who received semaglutide 1.0 mg or canagliflozin 300 mg using whole-body dual-energy x-ray absorptiometry (DXA).

## Methods

### SUSTAIN 8 study design

#### Trial design and participants

The trial design for SUSTAIN 8 (ClinicalTrials.gov registration no. NCT03136484) has been described previously [17]. Briefly, SUSTAIN 8 was a 52-week, Phase IIIB randomised double-blind, double-dummy, parallel-group trial of once-weekly semaglutide 1.0 mg vs once-daily canagliflozin 300 mg in 788 adults with type 2 diabetes on stable treatment with metformin. The trial was conducted at 111 centres in 11 countries. Adults (age ≥18 years) with uncontrolled type 2 diabetes, HbA_1c_ levels of 53–91 mmol/mol (7.0–10.5% [inclusive]) on a stable daily dose of metformin (≥1500 mg or maximum tolerated dose) for at least 90 days prior to screening and an eGFR ≥60 ml min^−1^ [1.73 m]^−2^ were eligible. Individuals with a history or presence of pancreatitis (acute or chronic), history of diabetic ketoacidosis, myocardial infarction, stroke, hospitalisation for unstable angina or transient ischaemic attack within 180 days prior to screening, and New York Heart Association class IV heart failure were excluded.

#### Randomisation and treatment

Participants were randomised in a 1:1 manner to receive either once-weekly semaglutide 1.0 mg subcutaneous injections and once-daily canagliflozin placebo oral tablets, or once-daily canagliflozin 300 mg oral tablets and once-weekly subcutaneous semaglutide placebo. Randomisation was stratified according to participation in the substudy (yes/no) to ensure balanced treatment allocation. Dosing for semaglutide began at 0.25 mg and doubled every 4 weeks until the maintenance dose of 1.0 mg was achieved at 8 weeks. Participants randomised to canagliflozin received 100 mg for 8 weeks, followed by an increase to the maintenance dose of 300 mg. The only background medication permitted was metformin (≥1500 mg or maximum tolerated dose). Participants continued on their pre-trial dose throughout the treatment period unless rescue medication was required. After a treatment period of 52 weeks, participants entered a 5-week follow-up period.

#### Outcomes

The primary and confirmatory secondary endpoints were change from baseline to week 52 in HbA_1c_ (%-point) and body weight (kg), respectively.

#### Ethics and consent

SUSTAIN 8 was conducted in compliance with the International Conference on Harmonisation Good Clinical Practice guidelines [25] and the Declaration of Helsinki [26]. The trial protocol was approved by the institutional review board and ethics committee at each participating centre, and participants provided written informed consent before trial-related activities commenced.

### DXA substudy

A planned subset of participants from the overall SUSTAIN 8 population received a DXA whole-body scan at baseline and were randomised for inclusion in the body composition substudy if the imaging laboratory deemed the quality of the scan to be acceptable.

#### Assessments and outcomes

The process of DXA scan image acquisition, transfer, central analysis, reporting of results and arching followed the charter prepared by one imaging laboratory (PAREXEL Informatics Medical Imaging, Waltham, MA, USA) where scans were analysed. GE Lunar iDXA (GE Healthcare, Madison, WI, USA) and the Hologic Discovery DXA system (Hologic, Marlborough, MA, USA) were used with Prodigy and APEX software, respectively. Each participant received one scan 5 days after the screening (visit 1) and a second, final scan 5 days after the end-of-treatment visit (planned study end or premature treatment discontinuation). The quality of the DXA scans was evaluated by the same imaging laboratory in a blinded manner.

Change from baseline to week 52 in total fat mass (kg) was the confirmatory efficacy endpoint in the DXA substudy. Changes from baseline to week 52 in total fat mass (%-point), total lean mass (kg and %-point), visceral fat mass (kg and %-point) and ratio of total fat mass to total lean mass (muscle mass in this study) were additional efficacy endpoints. A comparison of change in body weight within the substudy vs the primary study was performed post hoc in order to confirm that weight loss in individuals undergoing a DXA scan in the substudy was representative of the weight loss in the primary study.

#### Statistical analyses

The primary estimand was defined as the treatment difference between semaglutide and canagliflozin at week 52 for all randomised participants if all participants completed treatment and did not start rescue medication. The primary estimand was used to estimate the expected benefit from the initiation and continuation of semaglutide compared with canagliflozin, drawing inferences only from data collected before discontinuation of trial product or initiation of rescue medication. The estimand was based on the full analysis set of all randomised participants using post-baseline measurements up to and including week 52 from the ‘on-treatment without rescue medication’ observation period to support an efficacy evaluation. The analysis of all endpoints in the substudy was based on the subset of full analysis set participants who participated in the substudy (DXA analysis set); however, participants had only two DXA scans each, and not all of these were performed within the ‘on-treatment without rescue medication’ observation period (used for the confirmatory analysis). In individuals for whom DXA data were collected outside of this period, only baseline data were included in the analysis, and the corresponding end-of-treatment data were multiple imputed, as described below.

An ANCOVA with treatment and region as categorical effects and baseline measurements as a covariate was used to analyse values at week 52, and change from baseline estimates were adjusted according to the pooled baseline value to allow for comparison between treatment arms. Before analysis, missing data were imputed using observed data from participants within the same treatment group, using a regression model including region as categorical effect and baseline value as covariate. Rubin’s rules were used to combine the analysis results to draw inference [27]. Regions were defined as North America (USA and Canada); Region Europe (UK, Ireland and Sweden); or International Operations (Lebanon, Malaysia, Argentina, Mexico, Brazil and India).

**Fig. 1:**
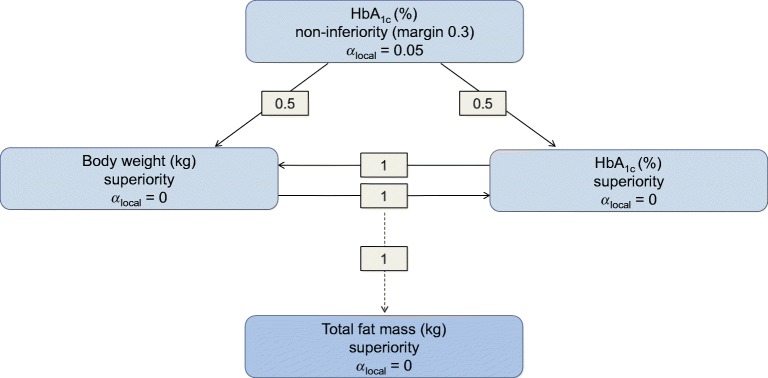
Graphical illustration of the closed testing procedure. The overall significance level of *α* = 0.05 (two-sided) is initially allocated to the HbA_1c_ non-inferiority test. The local significance level (*α*_local_) will be reallocated if a hypothesis is confirmed according to the weight given by the directed edges between nodes (hypotheses). The total fat-mass superiority test will receive the overall significance of *α* = 0.05 (two-sided) if, and only if, both HbA_1c_ and body weight superiority are confirmed at their respective local significance levels

As a confirmatory endpoint, change in total fat mass (kg) was tested for superiority of semaglutide 1.0 mg vs canagliflozin 300 mg. The overall type I error for the confirmatory hypotheses in SUSTAIN 8 and the substudy were controlled at a 5% level (two-sided) using a closed testing procedure (Fig. 1) [28]. Assuming a treatment difference of 1.8 kg and SD of 3.5 kg, it was estimated that 174 participants (87 per arm) would provide a 92% marginal power to establish a significant difference, resulting in 91% power for confirming superiority in the testing strategy for total fat mass loss (kg) at week 52.

Sensitivity analyses to evaluate the robustness of the conclusions from the confirmatory analysis (change in total fat mass [kg]) included a pre-specified in-trial analysis using all post-baseline measurements up to and including week 52 from the in-trial observation period, in which participants were considered to be in the trial after randomisation regardless of discontinuation of trial product or initiation of rescue medication. Scans that did not meet the criteria for inclusion in the confirmatory analysis (performed >7 days after last dose of trial product and thus considered out-of-window, or taken after initiation of rescue medication) were included in the in-trial supplementary analysis. Data from missing scans were multiple imputed.

In post hoc analyses, possible correlations between fat loss and changes in body weight, HbA_1c_ and BP were investigated. Correlations were calculated for each of the 500 complete multiple imputed datasets for body weight, HbA_1c_ and systolic and diastolic BP and combined using Rubin’s rules [27].

## Results

**Fig. 2:**
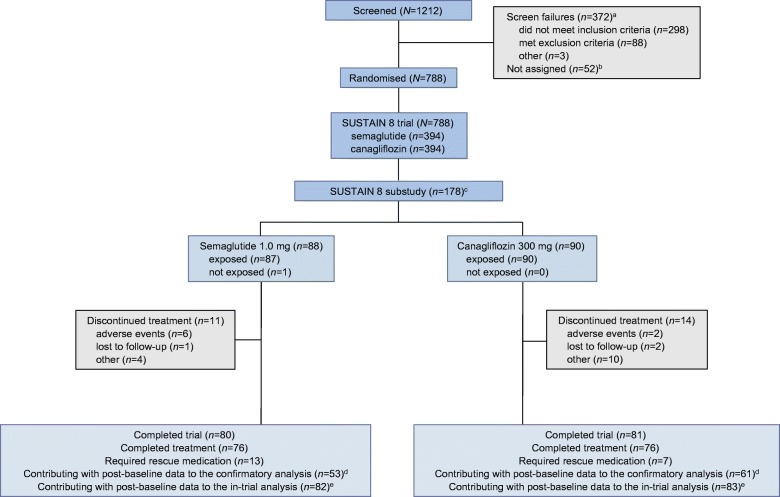
Participant disposition. ^a^Participants could meet more than one exclusion criterion. ^b^‘Not assigned’ includes individuals who withdrew consent before randomisation. ^c^Participants in the substudy were a subset of the overall SUSTAIN 8 trial population. ^d^In participants for whom data were outside the relevant observation period, only baseline participant data were included in the analysis, and the corresponding end-of-treatment data were multiple imputed. ^e^The in-trial analysis included all available post-baseline data. Missing data were multiple imputed

Between 15 March 2017 and 16 November 2018, 788 individuals were randomised to receive semaglutide 1.0 mg or canagliflozin 300 mg (each *n* = 394). A subset of 178 participants (semaglutide, *n* = 88; canagliflozin, *n* = 90) underwent DXA scanning at screening and were also randomised into the SUSTAIN 8 substudy. Of these, 177 (99.4%) participants (semaglutide, *n* = 87; canagliflozin, *n* = 90) were exposed to treatment. In total, 25 (14.0%) participants discontinued treatment prematurely, primarily because of adverse events (*n* = 8 [4.5%]). Overall, 165 of 178 participants (92.7%) had a post-baseline DXA scan available (Table 1; Fig. 2). Of these, data on 114 (64%: semaglutide, *n* = 53 [60.2%]; canagliflozin, *n* = 61 [67.8%]) were within the on-treatment without rescue medication period and used in the confirmatory efficacy analysis (Table 1; Fig. 2). The remaining post-baseline DXA scan results were excluded from the confirmatory analysis because scans at discontinuation were performed >7 days after the last dose of trial product (i.e. out-of-window; *n* = 38) or participants were on rescue medication at the time of the scan (*n* = 13). The pre-specified in-trial analysis used all post-baseline measurements up to and including week 52 from the in-trial observation period, i.e. all 165 available post-baseline scans (including out-of-window scans and scans of participants on rescue medication).

**Table 1:** Body composition substudy participant disposition and baseline characteristics

Variable	Semaglutide 1.0 mg (*n* = 88)	Canagliflozin 300 mg (*n* = 90)	Total (*n* = 178)
Participant disposition, *n* (%)			
Randomised, DXA	88 (100)	90 (100)	178 (100)
Exposed	87 (98.9)	90 (100)	177 (99.4)
Observed EOT scan in in-trial analysis^a^	82 (93.2)	83 (92.2)	165 (92.7)
Observed EOT scan in confirmatory analysis^b^	53 (60.2)	61 (67.8)	114 (64.0)
Treatment completers^c^	76 (86.4)	76 (84.4)	152 (85.4)
Without rescue medication	63 (71.6)	69 (76.7)	132 (74.2)
Premature treatment discontinuation^d^	11 (12.5)	14 (15.6)	25 (14.0)
Adverse events	6 (6.8)	2 (2.2)	8 (4.5)
Lost to follow-up	1 (1.1)	2 (2.2)	3 (1.7)
Other	4 (4.5)	10 (11.1)	14 (7.9)
Trial completers^e^	80 (90.9)	81 (90.0)	161 (90.4)
Demographics and baseline characteristics, mean (SD)			
Age, years	57.8 (9.9)	58.6 (10.1)	58.2 (10.0)
HbA_1c_, mmol/mol	69.4 (11.9)	66.7 (10.9)	68.0 (11.5)
HbA_1c_, %	8.5 (1.1)	8.3 (1.0)	8.4 (1.0)
Diabetes duration, years	8.8 (5.8)	8.5 (5.2)	8.7 (5.5)
Body weight, kg	89.0 (18.2)	87.6 (18.2)	88.3 (18.2)
BMI, kg/m^2^	32.6 (6.4)	32.3 (5.5)	32.4 (6.0)
Waist circumference, cm	104.0 (13.5)	105.9 (13.1)	105.0 (13.3)
Total fat mass,			
kg %	33.9 (11.9) 38.0 (8.4)	32.5 (10.0) 37.3 (7.3)	33.2 (11.0) 37.6 (7.8)
Total lean mass,			
kg %	51.3 (10.1) 59.1 (8.0)	51.3 (10.7) 59.7 (6.9)	51.3 (10.4) 59.4 (7.5)
Visceral fat mass,			
kg %	1.5 (0.8) 43.7 (16.2)	1.5 (0.8) 44.0 (15.3)	1.5 (0.8) 43.9 (15.7)
Total fat mass:total lean mass ratio	0.67 (0.23)	0.65 (0.20)	0.66 (0.22)

The baseline value is defined as the latest pre-dosing value

^a^Participants with end-of-treatment data included in the pre-specified supplementary analysis

^b^Participants with end-of-treatment data included in the pre-specified confirmatory analyses

^c^Participants who completed treatment according to the end-of-treatment form

^d^Includes only exposed participants

^e^Participants who completed the trial according to the end-of-trial form

EOT, end-of-treatment (planned or premature)

### Baseline characteristics

Baseline characteristics were comparable between treatment arms (Table 1). Participants had an overall mean (SD) baseline HbA_1c_ value of 68.0 mmol/mol (11.5) [8.4% (1.0)] and body weight of 88.3 kg (18.2). Overall mean (SD) baseline body composition values were 33.2 kg (11.0) and 37.6% (7.8) for total fat mass, 51.3 kg (10.4) and 59.4% (7.5) for total lean mass, and 1.5 kg (0.8) (representing 43.9% [15.7] of abdominal fat) for visceral fat mass. The overall mean (SD) baseline total fat mass to total lean mass ratio was 0.7 (0.2).

### Change in total fat mass

**Fig. 3:**
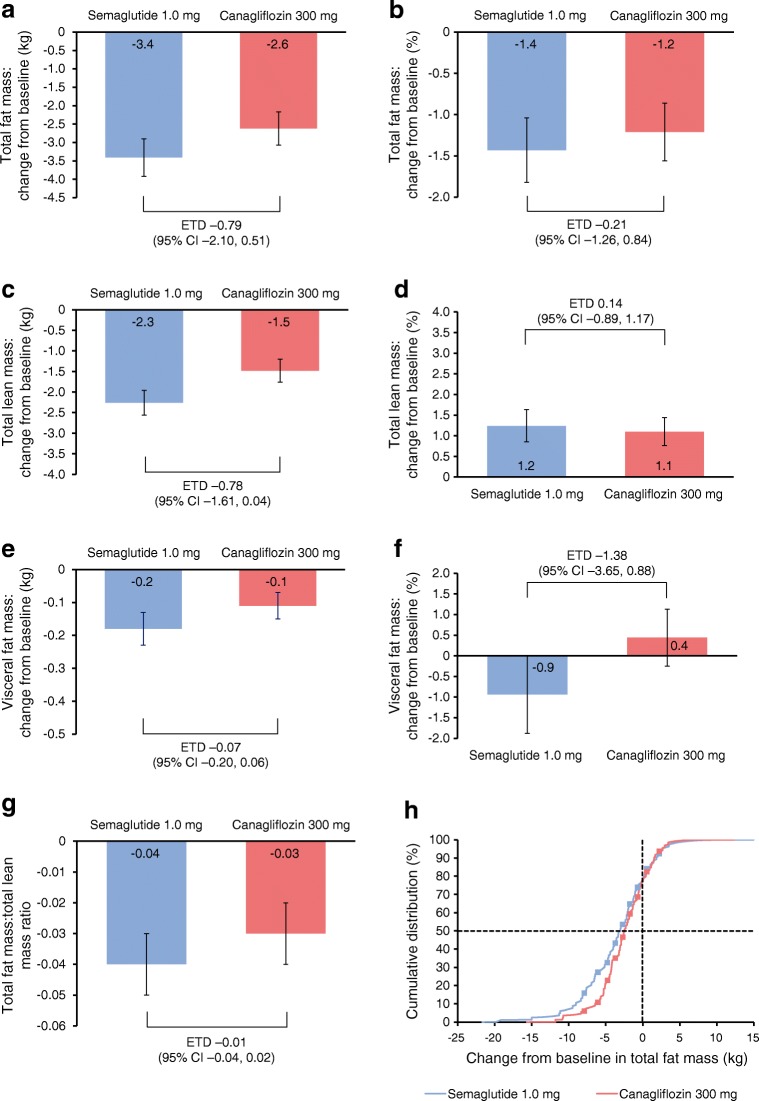
Body composition outcomes after 52 weeks of treatment. Change from baseline in total fat mass: kg (**a**) and % (**b**); total lean mass: kg (**c**) and % (**d**); visceral fat mass: kg (**e**) and % (**f**); ratio of total fat mass to total lean mass (**g**); and cumulative change in total fat mass (**h**). ‘On-treatment without rescue medication’ data ( *n* =114 [semaglutide, *n* = 53; canagliflozin, *n* = 61]). Missing data were multiple imputed using observed data from participants within the same group defined by randomised treatment, using a regression model including region as categorical effect and data from baseline as covariate. (**a**–**g**) Responses were analysed using an ANCOVA with treatment and region as fixed factors and baseline value as covariate. Regions were defined as North America (USA and Canada); Region Europe (UK, Ireland and Sweden); or International Operations (Lebanon, Malaysia, Argentina, Mexico, Brazil and India). Numbers on the bars may not match the numbers on the scale due to rounding. (**h**) The dashed line on the *y*-axis shows the 50th percentile, while the dashed line on the *x*-axis shows the reference value for no change

Numerical reductions in fat mass (kg and %-point) were observed with both treatments. Estimated reductions were numerically greater for semaglutide vs canagliflozin, but the differences were not statistically significant (Fig. 3a, b). Total fat mass (SE) was reduced with semaglutide and canagliflozin from an overall baseline of 33.2 kg: 3.4 kg (0.51) vs 2.6 kg (0.45), respectively (Fig. 3a), with an estimated treatment difference (ETD; 95% CI) of −0.79 (−2.10, 0.51). The supplementary in-trial analysis of all available data demonstrated similar, non-significant results for total fat mass with reductions of 3.0 kg (0.37) and 2.3 kg (0.36) for semaglutide and canagliflozin, respectively (ETD –0.71 [95% CI –1.72, 0.31]).

The proportion of total fat mass (SE) was reduced from an overall baseline of 37.6% by 1.4%-point (0.39) and 1.2%-point (0.35) with semaglutide and canagliflozin, respectively (ETD –0.21 [95% CI –1.26, 0.84]) (Fig. 3b). Cumulative changes in total fat mass were comparable between semaglutide and canagliflozin; over 52 weeks, approximately 80% of participants experienced reduced total fat mass with both treatments (Fig. 3h).

#### Post hoc correlation coefficient analysis

**Fig. 4:**
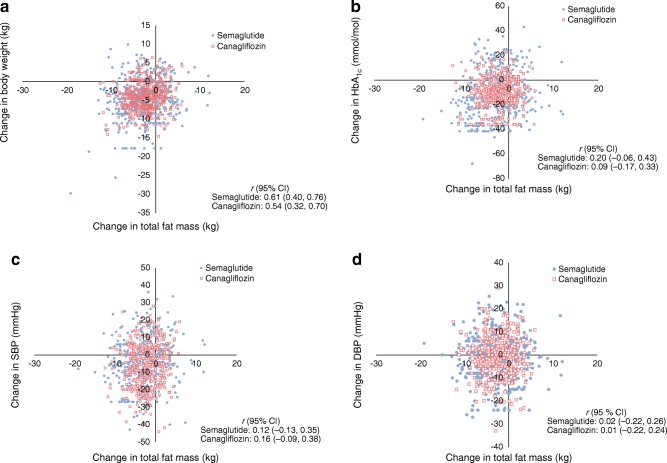
Correlation of absolute change in total fat mass (kg) with change in body weight (**a**), HbA_1c_ (**b**), SBP (**c**) and DBP (**d**) after 52 weeks of treatment. Each point represents data (observed or imputed) from one participant. ‘On-treatment without rescue medication’ data for all randomised participants (*n* = 178 [semaglutide, *n* = 88; canagliflozin, *n* = 90]). Missing data were multiple imputed using data from participants within the same group defined by randomised treatment. Data are plotted for the first ten out of 500 imputations. DBP, diastolic BP; SBP, systolic BP

A moderate correlation was observed between change in total fat mass and change in body weight between baseline and week 52 for semaglutide (*r* = 0.61 [95% CI 0.40, 0.76]) and canagliflozin (*r* = 0.54 [0.32, 0.70]) (Fig. 4a). There were no correlations between change in total fat mass and change in HbA_1c_, systolic BP or diastolic BP over the same period with either semaglutide or canagliflozin (*r =* 0.01–0.20) (Fig. 4b–d).

### Change in total lean mass

Total lean mass (SE) was reduced from an overall baseline of 51.3 kg by 2.3 kg (0.30) vs 1.5 kg (0.28) in the semaglutide and canagliflozin treatment groups, respectively (ETD –0.78 [95% CI –1.61, 0.04]) (Fig. 3c). In contrast, lean mass as a proportion of the whole (SE) increased from an overall baseline of 59.4% by 1.2%-point (0.39) with semaglutide vs 1.1%-point (0.34) with canagliflozin (ETD 0.14 [−0.89, 1.17]) (Fig. 3d).

### Change in visceral fat mass

The decreases in visceral fat mass (SE) were 0.2 kg (0.05) and 0.1 kg (0.04) with semaglutide and canagliflozin, respectively (ETD –0.07 [95% CI –0.20, 0.06]) (Fig. 3e). The percentage of visceral fat mass (SE) decreased from an overall baseline mean of 43.9% by 0.9%-point (0.94) with semaglutide vs an increase of 0.4%-point (0.69) with canagliflozin (ETD –1.38 [−3.65, 0.88]) (Fig. 3f).

### Ratio between total fat mass and total lean mass

Body composition changes, as assessed by the fat to lean mass ratio (SE), were favourable for both treatment groups: −0.04 (0.01) and −0.03 (0.01) with semaglutide and canagliflozin, respectively (ETD –0.01 [95% CI –0.04, 0.02]) (Fig. 3g).

### Change in body weight

**Fig. 5:**
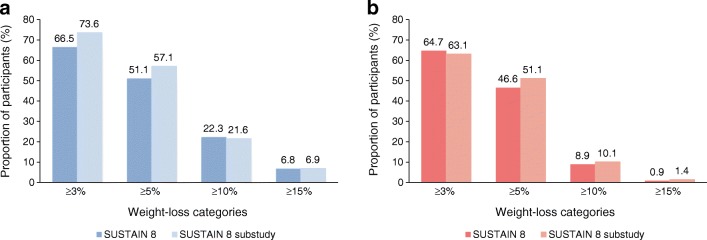
Proportion of participants achieving weight loss ≥3%, 5%, 10% or 15% of body weight from baseline after 52 weeks of treatment with semaglutide 1.0 mg (**a**) and canagliflozin 300 mg (**b**) in SUSTAIN 8 (*n* = 788) and the SUSTAIN 8 substudy (*n* = 178). ‘On-treatment without rescue medication’ data. Missing data were multiple imputed using observed data from participants within the same group defined by randomised treatment, using a regression model including region and stratification factor as categorical effects and data from baseline and all previous visits as covariates. Regions were defined as North America (USA and Canada); Region Europe (UK, Ireland and Sweden); or International Operations (Lebanon, Malaysia, Argentina, Mexico, Brazil and India). This post hoc comparison of change in body weight within the substudy vs the primary study was performed in order to confirm that weight loss in participants undergoing a DXA scan in the substudy was representative of the weight loss in the primary study

In a post hoc calculation of the substudy cohort (*n* = 178), which served to illustrate consistency between weight loss in participants undergoing a DXA scan in the substudy and in the primary study, reduction in total fat plus lean mass was 5.7 kg with semaglutide vs 4.1 kg with canagliflozin in the substudy, suggesting a weight-loss pattern similar to that observed between treatments in the whole SUSTAIN 8 population (Table 2). Moreover, the distribution of body weight loss was similar in the SUSTAIN 8 substudy compared with the main SUSTAIN 8 trial in participants receiving both semaglutide and canagliflozin (Fig. 5a, b, respectively).

**Table 2:** Body composition outcomes at week 52 (‘On-treatment without rescue medication’ data)

	Semaglutide 1.0 mg (*n* = 88)	Canagliflozin 300 mg (*n* = 90)	ETD (95% CI)
Total fat mass^a^			
Change at week 52, kg	−3.41 (0.51)	−2.62 (0.45)	−0.79 (−2.10, 0.51)
Change at week 52, %-point	−1.43 (0.39)	−1.21 (0.35)	−0.21 (−1.26, 0.84)
Total lean mass			
Change at week 52, kg	−2.26 (0.30)	−1.48 (0.28)	−0.78 (−1.61, 0.04)
Change at week 52, %-point	1.24 (0.39)	1.10 (0.34)	0.14 (−0.89, 1.17)
Total fat plus lean mass (post hoc assessment)^b^			
Change at week 52, kg	−5.7	−4.1	
Total fat mass:total lean mass, kg			
Change at week 52	−0.04 (0.01)	−0.03 (0.01)	−0.01 (−0.04, 0.02)
Visceral fat mass			
Change at week 52, kg	−0.18 (0.05)	−0.11 (0.04)	−0.07 (−0.20, 0.06)
Change at week 52, %-point	−0.94 (0.94)	0.44 (0.69)	−1.38 (−3.65, 0.88)
Waist circumference, cm			
Change at week 52^c^	−3.9 (5.6)	−2.5 (5.5)	

Data are mean (SE) unless otherwise specified and represent estimates from an ANCOVA with treatment, region and baseline value as fixed effects

Multiple imputation was used where missing data were imputed using observed data from participants within the same group defined by randomised treatment, using a regression model including region and stratification factor as categorical effects and data from baseline and all previous visits as covariates

Regions were defined as North America (USA and Canada); Region Europe (UK, Ireland and Sweden); or International Operations (Lebanon, Malaysia, Argentina, Mexico, Brazil and India)

^a^For total fat mass, responses were analysed with an ANCOVA with treatment and region as fixed factors and baseline value as covariate. Before analysis, missing data were multiple imputed using observed data from participants within the same group defined by randomised treatment, using a regression model including region as categorical effect and data from baseline as covariate

^b^Not pre-specified for the substudy; data are calculated as the sum of the estimated change (mean [SD])

^c^Not pre-specified for the substudy; data are mean (SD)

## Discussion

In the SUSTAIN 8 substudy, numerical reductions in absolute and proportion of total fat mass were observed for treatment with both semaglutide 1.0 mg and canagliflozin 300 mg. Although fat loss was numerically greater with semaglutide vs canagliflozin after 52 weeks of treatment, the difference was not statistically significant. Overall, 36% of participants were missing DXA scans for the end-of-treatment confirmatory analysis, mainly because scans were taken out-of-window (>7 days after the last dose of trial product). However, the supplementary in-trial analysis of all available scans demonstrated similar results, supporting the robustness of the conclusion for this endpoint. Numerical reductions were observed in both treatment groups in absolute lean mass (kg), although an increase in the proportion of total lean mass (%-point) was observed. However, the change in ratio between total fat mass and total lean mass was minimal in both treatment groups, and was not significantly different between groups. A marginal decrease in absolute visceral fat mass was observed with both treatments, whereas the proportion of visceral fat decreased slightly with semaglutide and increased slightly with canagliflozin, although differences between treatment arms were not significant. Importantly, in this substudy, there was no evidence of deleterious body composition changes, such as reductions in proportion of total lean mass; although the specific impact of both treatments on body composition in the absence of a placebo arm is speculative at this stage.

DXA, a two-compartment method of body composition analysis that separates body components into fat mass and fat-free mass, is one of the most popular and widely used non-invasive techniques for estimating whole-body and regional body composition [29]. When used in relation to body composition, the term ‘lean mass’ usually refers to muscle mass; however, lean mass comprises the combined weight of internal organs, muscle, connective tissue and water. It is important to consider the influence of these non-muscle components in DXA measurements of lean mass, and particularly the inability of DXA to calculate variable amounts of water [29], making it difficult to distinguish between loss of muscle and reduction in extracellular volume.

The link between obesity and type 2 diabetes is well known [1, 2]. Abdominal obesity is a key factor in the development of diabetes and cardiovascular disease [30] and is a risk factor for several chronic diseases with cardiometabolic homeostasis [31]. Recent studies showing reduction in visceral fat in normal-weight [31] and overweight individuals [32] may have implications with regard to improving cardiometabolic profile. Although many conventional glucose-lowering therapies may contribute to weight gain, treatment with GLP-1RAs and SGLT-2is has been associated with clinically meaningful weight loss [2]. In SUSTAIN 8, treatment with semaglutide and canagliflozin resulted in substantial weight loss of −5.3 kg and −4.2 kg, respectively (ETD –1.06 [95% CI –1.76, −0.36]; *p* = 0.003 favouring semaglutide), with more participants in the semaglutide arm achieving weight loss ≥10% than those in the canagliflozin arm (22.3% vs 8.9%; OR 2.99 [95% CI 1.89, 4.75]; *p* < 0.0001). A post hoc calculation of reduction in overall mass (fat mass plus lean body mass) in the DXA substudy indicated reductions of 5.7 kg vs 4.1 kg for semaglutide and canagliflozin, respectively. The distribution of body weight loss in participants in the SUSTAIN 8 substudy receiving semaglutide and canagliflozin was similar compared with all participants in the primary SUSTAIN 8 trial.

The magnitude of weight loss may have implications for the effect of treatment on lean muscle mass. Regular diet-induced weight loss is associated with corresponding changes in both body fat and fat-free mass, including muscle [33]. In individuals with type 2 diabetes, it is preferable to reduce fat without significant loss of lean mass [32], although this can occur during weight loss in those who are obese [34]. Because skeletal muscle is the major site of postprandial glucose uptake [35], a decrease in lean mass may be associated with impaired glucose metabolism in individuals with type 2 diabetes. However, this is a complex process for which there are various contributing factors. A recent trial examining the effect of the SGLT-2i ipragliflozin in Japanese people with obesity revealed similar mean reductions of fat mass and total lean mass of 1.8 kg and 1.7 kg, respectively, including a small but significant 0.6 kg loss in appendicular lean mass (a good marker of skeletal muscle mass), suggesting that SGLT-2is may induce catabolism of both body fat and muscle secondary to glycosuria [36]. However, the use of DXA for the estimation of changes in body composition in SGLT-2i trials has demonstrated greater reductions in fat mass (2.2 to 2.4 kg) vs lean mass (1.1 to 1.2 kg) [37, 38]. These reductions occurred in the context of a sustained elevation in spot urinary glucose excretion, which is associated with decreased total body weight and fat mass. Thus, the caloric loss from glucosuria, and not fluid loss, was responsible for the reductions [38]. This is supported by results from a small study finding that changes in extracellular water volume with SGLT-2is are transient, and not responsible for long-term weight loss with this drug class [39].

Similar results were observed in trials assessing body composition with other GLP-1RAs [32, 40, 41]. In a substudy of the LEAD-2 trial, liraglutide 1.8 mg led to changes from baseline in fat mass and lean mass of −2.4 kg and −1.5 kg, respectively, over 26 weeks in 37 participants with type 2 diabetes [40]. In a separate prospective case-series study investigating liraglutide in nine overweight or obese elderly individuals with uncontrolled type 2 diabetes on metformin, DXA demonstrated that mean fat mass was reduced by 1.5 kg, but skeletal muscle index (a measure of muscle mass) improved [41]. A second prospective case-series study evaluated liraglutide 0.9 mg once daily in obese Japanese individuals over 24 weeks [32]. In that study (*n* = 9), body weight was reduced by 11.7%. Body composition analysis from DXA scans showed that this was mostly caused by decreases in visceral fat mass (mean reduction: 11.9%) and intrahepatic lipid content (mean reduction: 49.2%) with no change in subcutaneous fat. Fat mass index also decreased (mean reduction: 10.9%) whereas skeletal muscle mass remained unchanged [32]. While indirect comparisons across clinical trials should be interpreted with caution, given the heterogeneity of the study populations, these findings suggest that body composition changes with semaglutide are consistent with those in previous studies of other GLP-1RAs. These changes are supported by the proposed mechanisms of weight loss with GLP-1RAs through appetite modulation via the central nervous system with minimal effect on energy expenditure [42, 43], as well as through the increase of natriuresis caused by inhibition of the sodium–hydrogen ion transporter in the proximal tubule, which subsequently reduces sodium retention and extracellular fluid volume expansion [44]. An additional mechanism may be nausea/vomiting, which was shown to have a minimal effect on weight loss in a post hoc mediation analysis of semaglutide vs exenatide extended release and dulaglutide in the SUSTAIN 3 and 7 trials [45].

The positive effects of both treatments on weight loss demonstrated previously [11–20, 24] highlight the importance of newer agents among current pharmacological options, compared with older, standard approaches for type 2 diabetes that may increase or have little effect on weight [46]. However, the greater reduction of weight loss with semaglutide vs canagliflozin in the primary SUSTAIN 8 trial [17], when also taking into account the lack of statistical difference in body composition changes between treatments in this substudy, highlights the potential value of semaglutide in terms of weight management in individuals with uncontrolled type 2 diabetes on stable-dose metformin therapy.

### Strengths and weaknesses

The strengths of this substudy include the methods and design of SUSTAIN 8, a double-blind randomised clinical trial with a global population, relatively long treatment period of 52 weeks, and relevant head-to-head comparison with a glucose-lowering medication with demonstrated efficacy in type 2 diabetes. In addition, analysis and quality assessments of the DXA scans were performed by one central imaging laboratory using one brand of scanner, reducing the likelihood of inconsistencies in bone and soft-tissue measurements that can occur due to variability of hardware and software packages between DXA equipment manufacturers [47].

DXA is one of the most popular and widely used non-invasive techniques for estimation of whole-body and regional body composition [47–49]. However, body thickness and hydration status can affect results [49], and lean muscle mass may also include non-muscle components, such as blood or interstitial fluid, leading to measurement error [50]. Moreover, the inability of DXA to distinguish different types of fat (visceral, subcutaneous, intramuscular) and lean soft tissues (muscle, organs) represents a practical limitation [49]. The lack of systematic collection of off-treatment DXA scans is a weakness of this substudy. Some participants only received baseline scans, and approximately a third did not have scans available for the confirmatory analysis, mostly due to these being taken out-of-window. However, results from the supplementary in-trial analysis, which included all available data, were similar to those observed in the confirmatory analysis and confirm the robustness of data. Finally, the SUSTAIN 8 trial did not include a placebo arm, meaning that all changes in body weight and composition reported here are comparisons between active treatments, and it cannot be concluded that all observed changes were entirely attributable to either treatment.

### Conclusion

In the SUSTAIN 8 substudy, the changes in body composition between semaglutide and canagliflozin were not significantly different in participants with uncontrolled type 2 diabetes on stable-dose metformin therapy. Although numerical improvements in body composition were observed following treatment in both treatment arms, the specific impact of both treatments on body composition in the absence of a placebo arm is speculative at this stage.

## Data Availability

Individual participant data will be shared in datasets in a de-identified/anonymised format. Datasets from Novo Nordisk-sponsored clinical research completed after 2001 for product indications approved in both the EU and US will be shared. The study protocol and redacted Clinical Study Report (CSR) will be available according to Novo Nordisk data sharing commitments. The data will be available permanently after research completion and approval of product and product use in both EU and US. There is no end date. Data will be shared with bona fide researchers submitting a research proposal and requesting access to data, for use as approved by the Independent Review Board (IRB) according to the IRB Charter (see www.novonordisk-trials.com). The access request proposal form and the access criteria can be found at www.novonordisk-trials.com. The data will be made available on a specialised SAS data platform.

## Abbreviations

DXA: Dual-energy x-ray absorptiometry
ETD: Estimated treatment difference
GLP-1RA: Glucagon-like peptide-1 receptor agonist
SGLT-2i: Sodium–glucose co-transporter-2 inhibitor
SUSTAIN: Semaglutide Unabated Sustainability in Treatment of Type 2 Diabetes
